# Supplementary material for: Analyzing the evolutionary trajectory of technological themes based on the BERTopic model: A case study in the field of artificial intelligence
Source: PLoS One. 2025 Jun 5;20(6):e0324933. doi: 10.1371/journal.pone.0324933 (PMC12140655; doi:10.1371/journal.pone.0324933)
Supplement: S3 Code — https://doi.org/10.6084/m9.figshare.28853594.v1. (PDF) [file pone.0324933.s003.pdf]

```
from sentence_transformers import SentenceTransformer
model = SentenceTransformer('sentence-transformers/paraphrase-multilingual-MiniLM-L12-v2')
import pandas as pd
import numpy as np
text = []
with open('word.txt','r',encoding='utf-8') as f:
    txt = f.readline()
    for txt in f:
        text.append(txt.strip())
df = pd.DataFrame({'segment':text})
import jieba
df['segment0'] = df['segment'].apply(lambda x: x.strip())
print("bertopic start operating")
from bertopic import BERTopic
from sentence_transformers import SentenceTransformer
from umap import UMAP
from sklearn.preprocessing import StandardScaler
import matplotlib.pyplot as plt
import pandas as pd
from hdbscan import HDBSCAN
import plotly.io as pio
import joblib
# Step 1 - Embed documents
embedding_model = SentenceTransformer('paraphrase-multilingual-MiniLM-L12-v2')
# Step 2 - Reduce dimensionality
umap_model = UMAP(n_neighbors=15, n_components=60, min_dist=0.01, metric='cosine')
# Step 3 - Cluster reduced embeddings
hdbscan_model = HDBSCAN(min_cluster_size=50, metric='euclidean', prediction_data=True)
# Step 4 - Create topic representation
from sklearn.feature_extraction.text import CountVectorizer
from bertopic.vectorizers import ClassTfidfTransformer
vectorizer_model = CountVectorizer(analyzer='word', token_pattern=u"(?u)\\b\\w+\\b")
ctfidf_model = ClassTfidfTransformer()
topic_model = BERTopic(
    embedding_model=embedding_model,        # Step 1 - Extract embeddings
    umap_model=umap_model,                  # Step 2 - Reduce dimensionality
    hdbscan_model=hdbscan_model,            # Step 3 - Cluster reduced embeddings
    vectorizer_model=vectorizer_model,      # Step 4 - Tokenize topics
    ctfidf_model=ctfidf_model,              # Step 5 - Extract topic words
    diversity=0.5,                          # Step 6 - Diversify topic words
    nr_topics='auto',
    top_n_words = 10
)
filtered_text = df["segment0"].tolist()
```

```
topics, probabilities = topic_model.fit_transform(filtered_text)
print(filtered_text[0])
document_info = topic_model.get_topic_info()
#print(document_info)
topic_freq = topic_model.get_topic_freq()
print(topic_freq)
#topics_info = topic_model.get_topic()
topic = topic_model.get_topic(0)
print(topic)
document_info_df = pd.DataFrame(document_info, columns=['Topic', 'Count', 'Name'])
topic_freq_df = pd.DataFrame(topic_freq, columns=['Topic', 'Count'])
topics_info_df = pd.DataFrame(topic, columns=['Words', 'Probabilities'])
#topics_info_df = pd.DataFrame(columns=['Topic', 'Words', 'Probabilities'])
#for topic_index, (words, probabilities) in enumerate(topics_info):
    #topics_info_df = topics_info_df.append({'Topic': topic_index, 'Words': words, 'Probabilities': probabilities}, ignore_index=True)
pic_bar = topic_model.visualize_barchart()
pio.show(pic_bar)
embeddings = embedding_model.encode(filtered_text, show_progress_bar=False)
# Run the visualization with the original embeddings
pic_doc = topic_model.visualize_documents(filtered_text, embeddings=embeddings)
pio.show(pic_doc)
pic_hie = topic_model.visualize_hierarchy()
pio.show(pic_hie)
pic_heat = topic_model.visualize_heatmap()
pio.show(pic_heat)
pic_topics = topic_model.visualize_topics()
pio.show(pic_topics)
pic_term_rank = topic_model.visualize_term_rank()
pio.show(pic_term_rank)
#joblib.dump(topic_model, 'bertopic_model.pkl')
```
