# Supplementary material for: Analyzing the evolutionary trajectory of technological themes based on the BERTopic model: A case study in the field of artificial intelligence
Source: PLoS One. 2025 Jun 5;20(6):e0324933. doi: 10.1371/journal.pone.0324933 (PMC12140655; doi:10.1371/journal.pone.0324933)
Supplement: S4 Code — https://doi.org/10.6084/m9.figshare.28853555.v1. (PDF) [file pone.0324933.s004.pdf]

```
from gensim import corpora, models
from gensim.models import Word2Vec
from gensim.similarities import Similarity
import logging
import cmath
from sklearn.decomposition import PCA
from matplotlib import pyplot
import numpy as np
def infile(fliepath):
    train = []
    fp = open(fliepath,'r',encoding='utf8')
    for line in fp:
        line = line.strip().split(' ')
        train.append(line)
    return train
sentences=infile('word2vec_training.txt')
model=models.Word2Vec(sentences,min_count=5)
print(model)
model.save('w2v.model')
```
