# Supplementary material for: Analyzing the evolutionary trajectory of technological themes based on the BERTopic model: A case study in the field of artificial intelligence
Source: PLoS One. 2025 Jun 5;20(6):e0324933. doi: 10.1371/journal.pone.0324933 (PMC12140655; doi:10.1371/journal.pone.0324933)
Supplement: S5 Code — https://doi.org/10.6084/m9.figshare.28853495.v1. (PDF) [file pone.0324933.s005.pdf]

```
# coding='utf-8'
from gensim import corpora, models
from gensim.models import Word2Vec
import math
from sklearn.decomposition import PCA
import json
def work(list_1, list_2):
    # x 值
    xs = []
    # y 值
    ys = []
    for i in list_1:
        xs.append(i[0])
        ys.append(i[1])
    for i in list_2:
        xs.append(i[0])
        ys.append(i[1])
    a = 0
    b = 0
    c = 0
    for x, y in zip(xs, ys):
        a = a + x * y
        b = b + x * x
        c = c + y * y
    h = a / (math.sqrt(b) * math.sqrt(c))
    return h.real
def infile(fliepath):
    train = []
    fp = open(fliepath, 'r', encoding='utf8')
    for line in fp:
        line = line.strip().split(' ')
        train.append(line)
    return train
sentences = infile('time_word.txt')
model = Word2Vec.load('w2v.model')
X = model.wv.vectors
pca = PCA(n_components=2)
result = pca.fit_transform(X)
words = list(model.wv.key_to_index)
'''
for i, word in enumerate(words):
    if word=='肺炎':
        print(word,result[i, 0], result[i, 1])
for sentence in sentences:
```

```
        print(sentence)
        for sen in sentence:
                print(sen)
'''
list_1 = []
for sentence in sentences:
        list_2 = []
        for sen in sentence:
                for i, word in enumerate(words):
                        if word == sen:
                                # print(word,result[i, 0], result[i, 1])
                                list_2.append((result[i, 0], result[i, 1]))
        list_1.append(list_2)
# print(len(list_1))
corpus = list_1
T1 = list(range(0, 10))
T2 = list(range(10, 25))
T3 = list(range(25, 45))
hs = {}
for i in T2:
        for j in T3:
                hs['T2 的主题词' + str(i) + str(sentences[i]) + '与' + 'T3 的主题词' + str(j - 25) + 
str(sentences[j]) + '的余弦相似度为'] = work(
                        corpus[i], corpus[j])
# print(hs)
for key, value in hs.items():    #
        print(key, '\t', value, '\n')
with open('T2-T3.json', 'w') as f:
        f.write(json.dumps(hs, ensure_ascii=False, indent=4, separators=(',', ':')))
print('保存成功')
```
